# Differences in verbal and spatial working memory in patients with bipolar II and unipolar depression: an MSI study

**DOI:** 10.1186/s12888-021-03595-3

**Published:** 2021-11-15

**Authors:** Zhinan Li, Junhao Chen, Yigang Feng, Shuming Zhong, Shui Tian, Zhongpeng Dai, Qing Lu, Yufang Guan, Yanyan Shan, Yanbin Jia

**Affiliations:** 1 Psychiatric Department, The First Affiliated Hospital of Jinan University, 613 West Huangpu Avenue, Guangzhou, 510630 China; 2 Psychiatric Department, The Third Affiliated Hospital of Sun Yat-sen University, Guangzhou, China; 3 Department of Electrophysiology, the Guangdong 999 brain Hospital, Guangzhou, China; 4 School of Biological Sciences & Medical Engineering, Southeast University, Nanjing, China; 5 Key Laboratory of Child Development and Learning Science, Southeast University, Nanjing, China

**Keywords:** Depression, Working memory, Frontoparietal network, Magnetoencephalography

## Abstract

**Background:**

Depressive symptoms could be similarly expressed in bipolar and unipolar disorder. However, changes in cognition and brain networks might be quite distinct. We aimed to find out the difference in the neural mechanism of impaired working memory in patients with bipolar and unipolar disorder.

**Method:**

According to diagnostic criteria of bipolar II disorder of the Diagnostic and Statistical Manual of Mental Disorders, Fifth Edition (DSM-5) and assessments, 13 bipolar II depression (BP II), 8 unipolar depression (UD) patients and 15 healthy controls (HC) were recruited in the study. We used 2-back tasks and magnetic source imaging (MSI) to test working memory functions and get the brain reactions of the participants.

**Results:**

Compared with HC, only spatial working memory tasks accuracy was significantly worse in both UD and BP II (*p* = 0.001). Pearson correlation showed that the stronger the FCs’ strength of MFG-IPL and IPL-preSMA, the higher accuracy of SWM task within left FPN in patients with UD (*r* = 0.860, *p* = 0.006; *r* = 0.752, *p* = 0.031). However, the FC strength of IFG-IPL was negatively correlated with the accuracy of SWM task within left FPN in patients with BP II (*r* = − 0.591, *p* = 0.033).

**Conclusions:**

Our study showed that the spatial working memory of patients with whether UD or BP II was impaired. The patterns of FCs within these two groups of patients were different when performing working memory tasks.

## Introduction

Up to 60% of patients with bipolar disorder are misdiagnosed as unipolar disorder what may considerably increase the risks of switching to suicide and poorer treatment responses [1]. Bipolar and unipolar disorder are sometimes confused in clinical practice which both could be manifested as symptoms of depression. Additionally, depressive symptoms may be either a risk factor or prodrome for cognitive deficits [2]. Cognitive deficit is one of the common symptoms of both bipolar and unipolar depression, even seems to be present in individuals in the remitted state [3, 4]. Furthermore, working memory is the basis for individuals to successfully perform various cognitive tasks, also plays an important role in complex cognitive activities [5]. Our and other’s previous studies suggested that decreased working memory performance in bipolar and unipolar disorder reflected neurofunctional deficits [6, 7].

The brain oscillations integrate functional brain systems across multiple spatiotemporal scales. Gamma oscillation (30–120 Hz) is a significant indicator of the functional activity of neurons. Many researchers found gamma oscillation was instrumental to the process of working memory [8]. Roux et al. [9] used magnetoencephalography (MEG) and a delayed match-to-sample task and found that there was a linear relationship between working memory-load and gamma oscillation activity in the prefrontal cortex, as the increased difficulty of working memory task, the activity also increased. Further, they suggested that gamma oscillation as well is involved in the maintenance of working memory information [10]. The findings of Haegens et al. supported the views of Roux et al., who found that gamma oscillation significantly increased during working memory tasks especially information coding and information retention in healthy control, besides they observed a significant positive correlation between gamma activity and task performance [11].

The frontoparietal network (FPN) includes portions of lateral precuneus, dorsolateral prefrontal cortex (DLPFC), and ventrolateral prefrontal cortex (VLPFC), and is associated with externally-oriented and cognitively demanding mental activity [12]. On the one hand, patients with bipolar disorder presented abnormal activation in regions typically involved in the FPN, according to a meta-analysis [13]. A study investigating multiple networks in euthymic bipolar I patients also identified an aberrantly increased connectivity in the FPN [14]. On the other hand, a meta-analysis identified that reduced connectivity within FPN and imbalanced connectivity with other networks involved in internal or external attention may reflect depressive biases toward internal thoughts at the cost of engaging with the external world in unipolar disorder patients [15]. Activation within FPN could be obviously observed during cognitive tasks, researchers believed that FPN contributed to functions such as working memory, cognitive control, and decision-making processes, moreover might be associated with regulation of emotion [16, 17]. Some evidence indicated that verbal working memory (VWM) activation mainly implicates left FPN, while the right FPN is associated with spatial working memory (SWM) [18]. However, more studies concluded that the completion of SWM has resulted in both two sides of FPN [19].

However, the results on working memory in depression are very heterogeneous, some studies believed that working memory was not impaired in bipolar or unipolar depression patients [20, 21]. It is perhaps due to different stimulation of the experimental paradigm is inconsistent. To avoid it, a 2-back task in which stimulus presentation and required response matched was used to compare the impaired condition of verbal and spatial working memory. Simultaneously, we used MEG to explore the neuroelectrophysiological mechanism between working memory deficits and gamma oscillation within FPN.

## Methods

### Subjects

We recruited patients with unipolar depression (UD) and patients with bipolar II depression (BP II) from the Psychiatry Department of the First Affiliated Hospital of Jinan University, Guangzhou, China. For each patient, the diagnosis was made according to the Diagnostic and Statistical Manual of Mental Disorders, Fifth Edition (DSM-5) by two experienced psychiatrists (Y.J. and S.Z.). 24-item Hamilton Depression Scale (HAMD) and the Young Mania Rating Scale (YMRS) were used to assessing the clinical state of each patient. The inclusion criteria were: (1) age 18–45 years, (2) experiencing a current episode of depressive symptoms, HAMD score > 20 and YMRS score < 8, (3) ≥ 9 years of education, and (4) right-handed with no experience of being left-handed. Drug screening was performed, and all the patients were either medication-naive or un-medicated for at least 2 months before this study. The exclusion criteria for the patients were as follows: (1) any Axis I disorders other than BP II and UD, (2) any history of organic brain disorders, (3) neurological illness, (4) mental retardation, cardiovascular diseases or alcohol/substance abuse, (5) a history of electroconvulsive therapy, and (6) severe myopia. Additionally, healthy controls (HC) were recruited via local advertisements. The exclusion criteria for the HC were the same as for the patients with the addition that they could not have had any history of psychiatric illness, any first-degree relatives with psychiatric illness, nor a significant medical or neurological illness either currently or previously. All the subjects satisfied the criteria for undergoing MEG recording and magnetic resonance imaging (MRI) scanning.

### 2-back working memory tasks

**Fig. 1:**
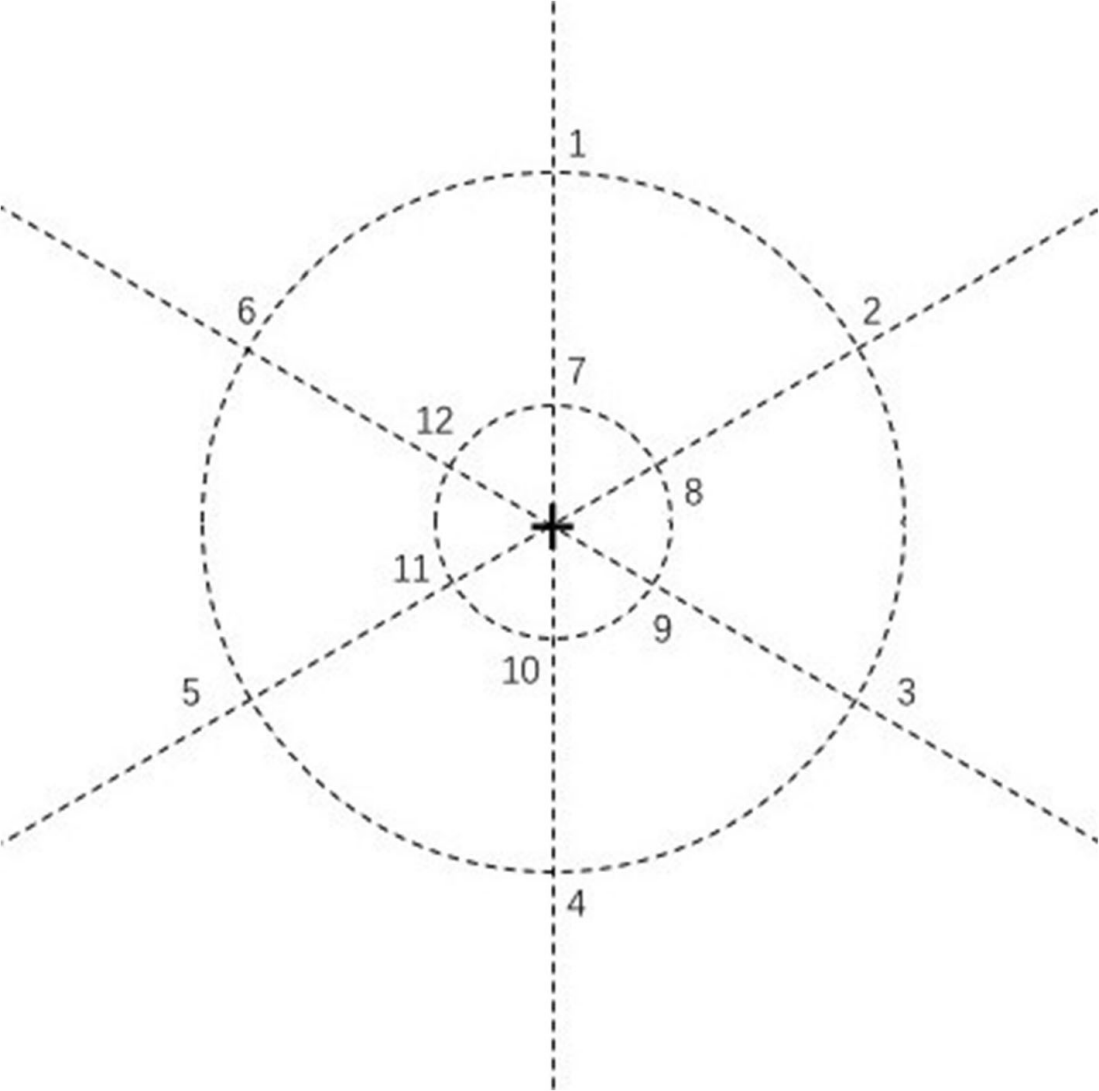
Possible locations of letters

**Fig. 2:**
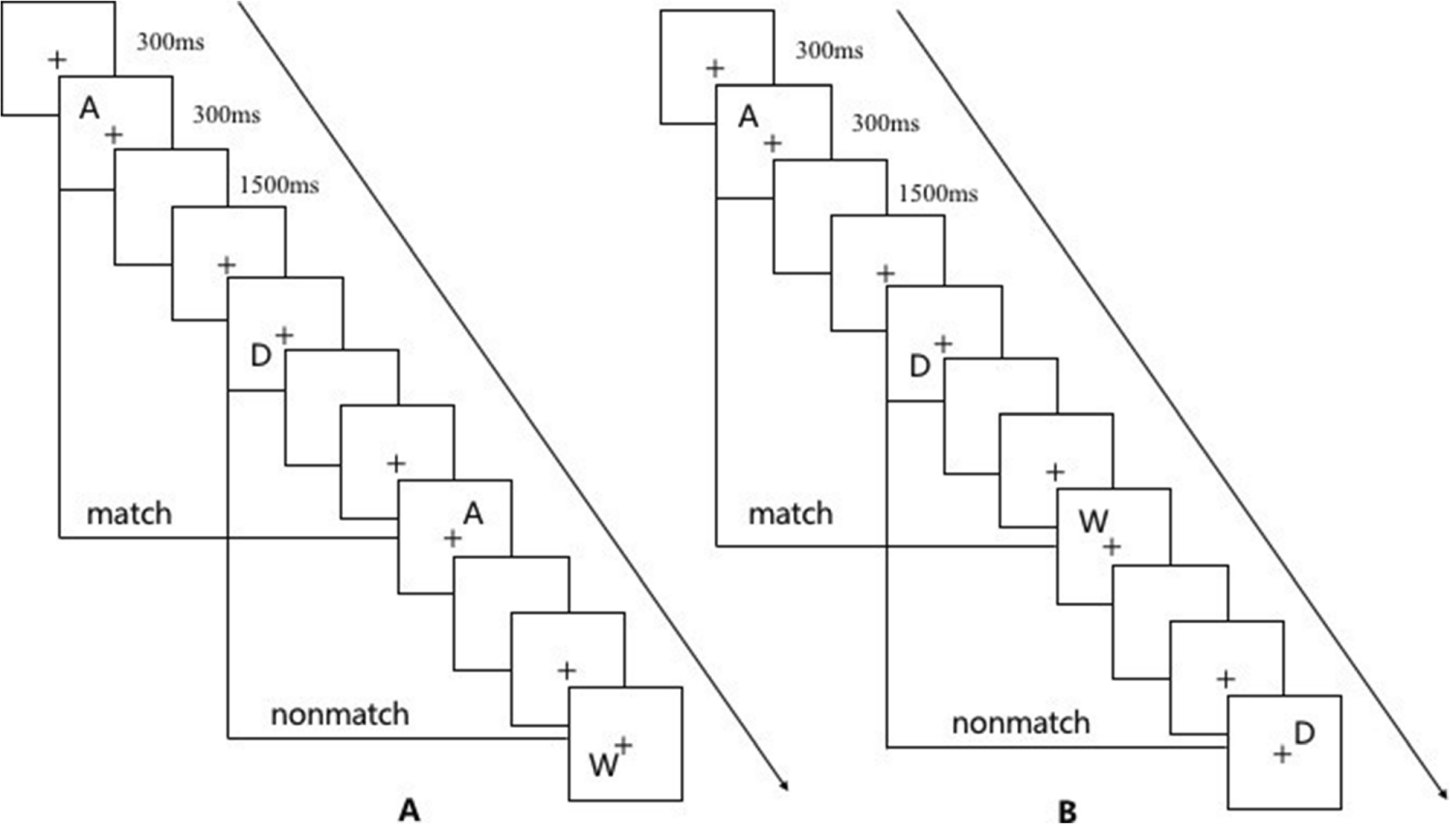
Depiction of 2-back working memory task. (A) verbal working memory; (B) spatial working memory

Based on the Li et al’s research [22], our tasks removed the pre-emotional induction. Black letter stimuli and a central fixation cross on a white background were presented on the computer. In the 2-back tasks, 12 possible letters (A, D, H, I, O, R, S, T, U, W, X, Y) were presented in one of 12 randomly predetermined screen locations (Fig*.* 1). Each of the 2-back tasks consisted of three blocks of 40 trials, with each trial was 2100 ms in duration, which was beginning with a central fixation cross for 300 ms, letter stimuli appeared for 300 ms, with the blank pages present through the 1500-ms-intertrial interval. In the verbal 2-back task, participants were instructed to respond with the “yes” key whenever the current letter stimulus matched the letter stimulus presented two trials previous in the same block, otherwise the “no” key was the correct response. The spatial location of the letter stimuli was task-irrelevant in the verbal tasks. In the spatial 2-back task, “yes” key responses were required whenever the current letter stimulus location matched the location of the letter stimulus two trials previous, otherwise, a “no” key response was required (Fig. 2). In all tasks, the probabilities of congruent trials were at 1/3, and errors were collected for each trial. Short breaks were provided between trial blocks in all sessions.

### MEG recordings

The MEG recordings were obtained in a magnetically shielded room using a 148-channel whole-head system (4D Neuroimaging, San Diego, CA) at a sampling rate of 1014.17 Hz, a band-pass filter specified as 0.1–200 Hz. Subjects were not allowed to possess any piece of metal that could cause magnetic artifacts. During the data sampling, each subject was asked to lie comfortably in a positive supine, rest their limbs, avoid moving their head, and complete 2-back tasks. Before initiating data acquisition, three electromagnetic coils were attached to reference landmarks on the left and right external acoustic meatuses and the nasion of each participant to check the head position. Head position moves exceeding approximately 5 mm were excluded. The recording session lasted for 450 s and was repeated twice. Subjects were monitored continuously via both camera and microphone.

### MRI scan

Structural MRI of all participants were scanned in a 1.5 T MRI (Philips Medical Systems, Netherlands) using a high-resolution, T1-weighted, 3D gradient-echo pulse sequence: field of view (FOV) = 240 × 240 mm, acquisition matrix =256 × 256, spin-echo repetition time (TR) =25 ms, echo time (TE) =4.6 ms, flip angle = 30°, slice thickness = 1.2 mm, slice gap = 0 mm). The offline MEG and MRI co-registration was performed using the fiducial markers (nasion, left and right external acoustic meatuses) by a manual method.

### Data processing

The MEG data were preprocessed with the Fieldtrip toolbox (fieldtrip.fcdonders.nl), via a band-stop filter to remove power-line interference (49.5–50.5 Hz) and a band-pass filter with 1-100 Hz cut-off. The deviated trials and channels were removed. The independent components that were produced by a temporal independent component analysis were visually checked to remove artifacts related to breathing, heartbeat, and muscle movement. After filtering to the gamma frequency band (45-60 Hz), source reconstruction of MEG data was conducted using the Fieldtrip toolbox. We selected regions of interest (ROIs) that correspond to those in previous studies as the most typical working memory-related regions in the FPN [19, 23]. Functional connectivity was then acquired by calculating the envelopment analysis of each paired ROI’s signal.

### Statistical analyses

Statistical analyses were implemented using the software SPSS version 23.0 (IBM, Inc.). A one-way analysis of variance (ANOVA) was used to test group differences in age, years of education, and FCs’ strength. A χ2 test was used to compare group differences in gender. A two-sample t-test was used to compare differences in clinical indices, such as disease duration. A Repeated Measures ANOVA was used to compare differences in groups (UD vs. BP II vs. HC) and working memory tasks accuracy (VWM vs. SWM). The correlations between clinical characteristics and working memory tasks accuracy were analyzed using Pearson correlation coefficients. The threshold of statistical significance for differences was set at *p* < 0.05 for each test. A post-hoc ANOVA showed a difference between the three groups to identify the significance of pair-wise group (BP II vs. UD, BP II vs. HC, and UD vs. HC) differences (p < 0.05, least significant difference test for multiple comparisons, Bonferroni).

## Results

### Clinical characteristics

This study included a total of eight patients diagnosed with UD, thirteen patients diagnosed with BP II, and fifteen HC. The BP II, UD, and HC groups were comparable in age, gender, and other characteristics including years of education, disease duration, and scale scores. The details are shown in Table 1.

**Table 1:** Characteristics of participants

Characteristic	HC (*n* = 15)	UD (*n* = 8)	BP II (*n* = 13)	*P* Value
Male gender (%) ^a^	7 (47)	5 (63)	4 (31)	0.254
Age (years) ^b^	23.80 ± 3.03	23.75 ± 5.65	23.31 ± 5.87	0.960
Years of education (years) ^b^	15.60 ± 1.30	14.88 ± 1.64	14.62 ± 2.66	0.404
Disease duration (months) ^c^	N. A.	14.00 ± 2.45	15.08 ± 6.567	0.601
HAMD score ^c^	N.A.	23.50 ± 4.90	24.00 ± 4.85	0.822
YMRS score ^c^	N.A.	1.38 ± 1.77	3.00 ± 2.71	0.149

All data were expressed as mean ± SD. ^a^ χ^2^ test, ^b^ one-way analysis of variance, ^c^ two-sample *t* test.

*HC* healthy control, *UD* unipolar depression, *BP II* bipolar II depression, *HAMD* Hamilton Depression Scale, *YMRS* Young Mania Rating Scale

### Working memory tasks accuracy

**Fig. 3:**
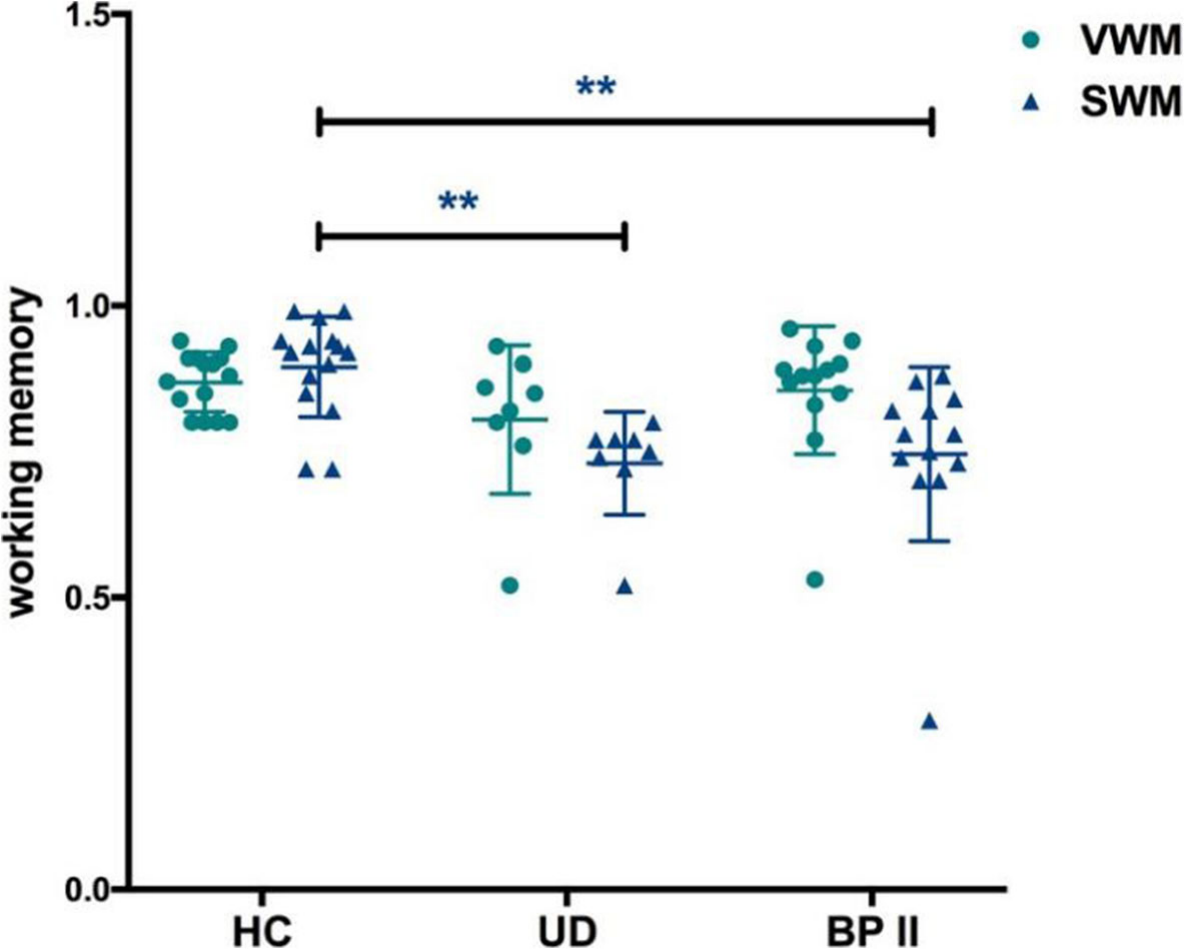
Verbal and spatial working memory tasks accuracy among 3 groups. VWM: verbal working memory; SWM: spatial working memory. HC: healthy control; UD: unipolar depression; BP II: bipolar II depression. *p < 0.05; **p < 0.01

For repeated measurement ANOVA, the interaction was significant (*F* = 11.420, *p* < 0.001). However, only the SWM tasks accuracy of UD and BP II patients were significantly worse than that of HC (*F* = 8.269, *p* = 0.001), but there was no significant difference between UD and BP II in Bonferroni post-hoc test (Fig. 3). In the VWM task, patients did not perform worse than HCs (*F* = 1.271, *p* = 0.294). We found no association between working memory tasks accuracy and characteristics (*p* > 0.05).

### Altered FCs within FPN during the spatial working memory task

Tables 2 and 3 exhibited the FCs that had significant differences among all groups during the SWM task. Results showed that FCs of left MFG-IPL, left IFG-IPL, and right MFG-SPL, right IPL-preSMA were weaker in patients with UD after Bonferroni correction. Furthermore, only FC of left IPL-preSMA was stronger in patients with BP II. However, during the VWM task, we found that the connectivity patterns of the three groups were similar (*p* > 0.05).

**Table 2:** Altered FCs within left FPN during spatial working memory task

Altered connectivity	HC (*n* = 15)	UD (*n* = 8)	BP II (*n* = 13)	*P* Value	post-hoc (*P* Value)
MFG-IFG	0.049 ± 0.008	0.048 ± 0.008	0.055 ± 0.007	0.049^*^	–
MFG-IPL	0.054 ± 0.007	0.047 ± 0.008	0.056 ± 0.009	0.032^*^	BP II, HC > UD
IFG-IPL	0.054 ± 0.006	0.050 ± 0.007	0.059 ± 0.007	0.012^*^	BP II, HC > UD
IPL-preSMA	0.050 ± 0.007	0.056 ± 0.010	0.058 ± 0.008	0.022^*^	BP II, UD > HC

All data were expressed as mean ± SD.

*HC* healthy control, *UD* unipolar depression, *BP II* bipolar II depression, *MFG* middle frontal gyrus, *IFG* inferior frontal gyrus, *preSMA* pre-supplementary motor area, *IPL* inferior parietal lobule.

*p < 0.05; **p < 0.01

**Table 3:** Altered FCs within right FPN during spatial working memory task

Altered connectivity	HC (*n* = 15)	UD (*n* = 8)	BP II (*n* = 13)	*P* Value	post-hoc (*P* Value)
MFG-SPL	0.054 ± 0.008	0.049 ± 0.004	0.060 ± 0.007	0.005^**^	BP II, HC > UD
IFG-IPL	0.055 ± 0.005	0.055 ± 0.008	0.062 ± 0.008	0.030^*^	–
IPL-preSMA	0.052 ± 0.008	0.047 ± 0.007	0.059 ± 0.009	0.009^**^	BP II, HC > UD

All data were expressed as mean ± SD.

*HC* healthy control, *UD* unipolar depression, *BP II* bipolar II depression, *MFG* middle frontal gyrus, *IFG* inferior frontal gyrus, *preSMA* pre-supplementary motor area, *IPL* inferior parietal lobule, *SPL* superior parietal lobule.

^*^p < 0.05; ^**^p < 0.01

### ROC curve analysis

**Fig. 4:**
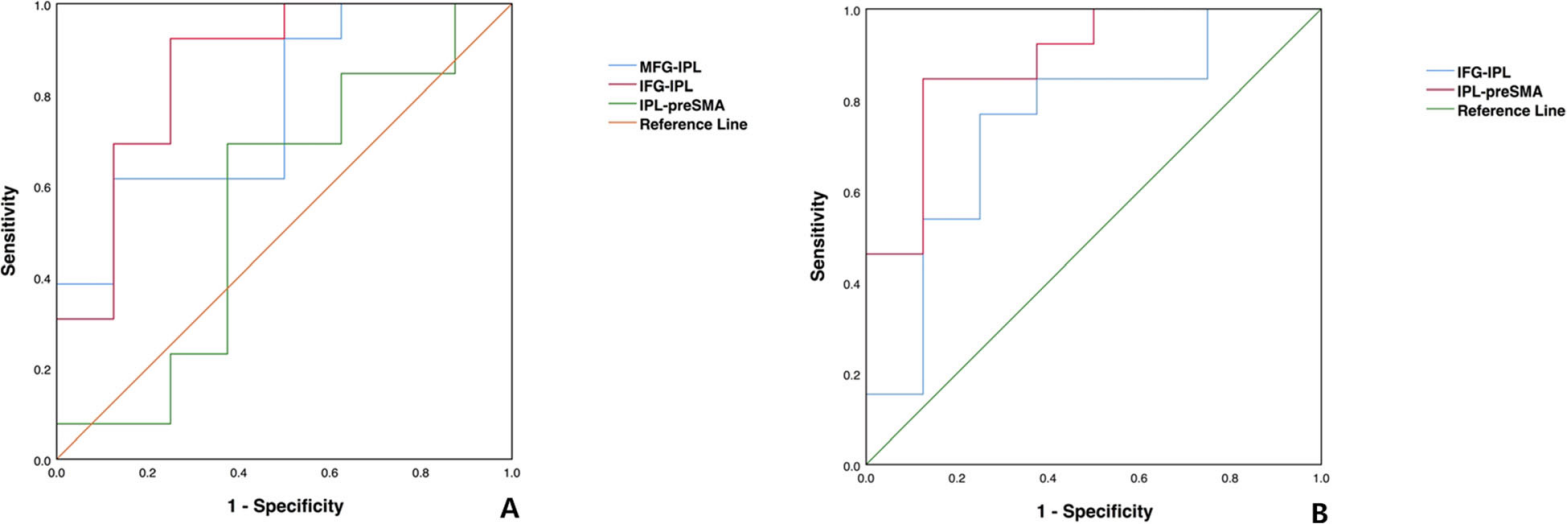
ROC curve analysis depicts the predictive performance of BP II and UD. (A) FCs of left FPN and (B) FCs of right FPN. MFG: middle frontal gyrus; IFG: inferior frontal gyrus; preSMA: pre-supplementary motor area; IPL: inferior parietal lobule

The predictive performance of FCs were shown in Fig. 4. The areas under the curve (AUC) for MFG-IPL, IFG-IPL, and IPL-preSMA within left FPN were 0.769, 0.856, and 0.558. The right IFG-IPL and IPL-preSMA were 0.750 and 0.855.

### Correlation between connectivity and working memory tasks accuracy

**Fig. 5:**
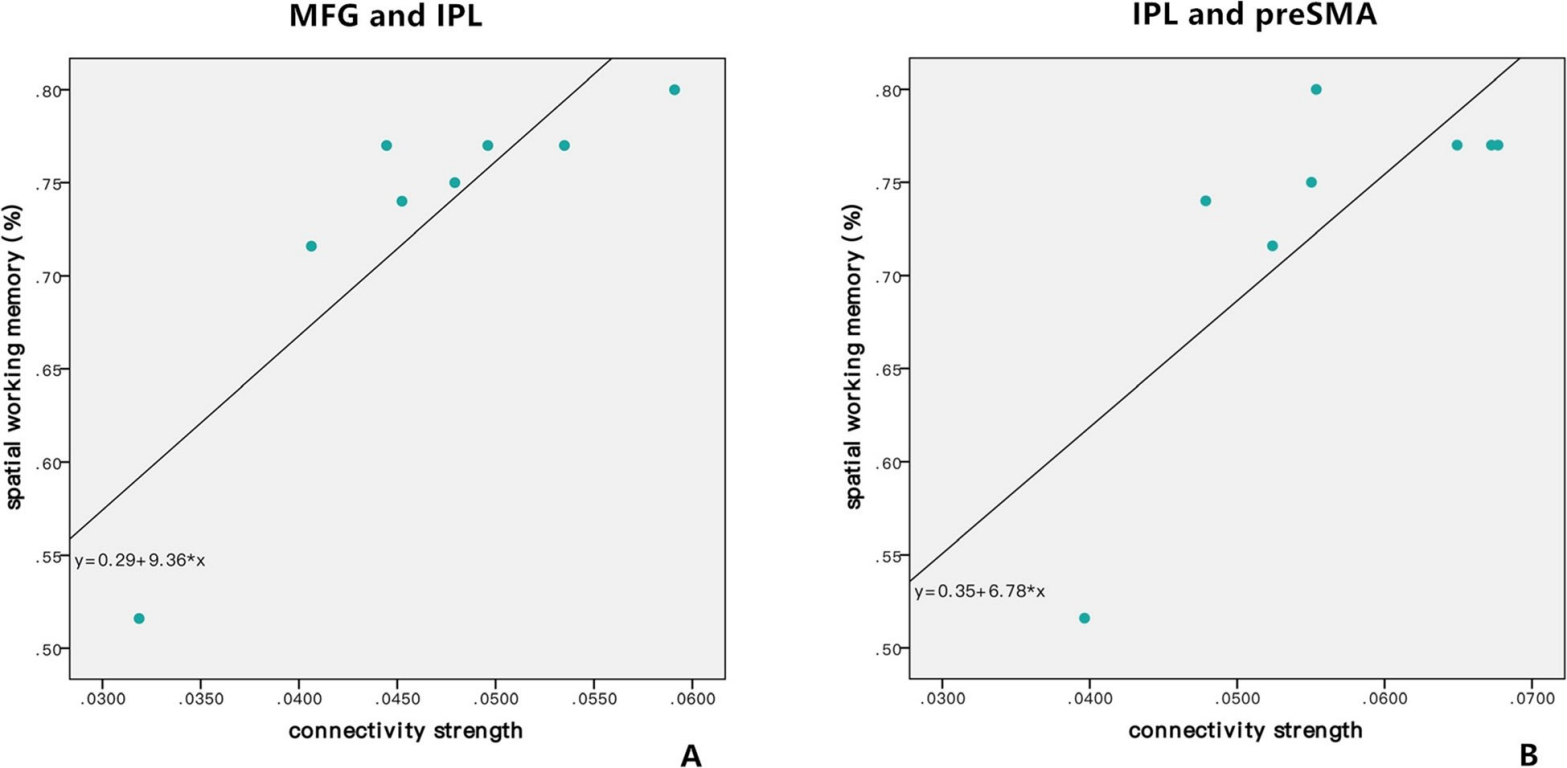
Correlation between SWM and FCs within left FPN in UD. (A) FC of MFG and IPL; (B) FC of IPL and preSMA. MFG: middle frontal gyrus; pre-SMA: pre-supplementary motor area; IPL: inferior parietal lobule; SWM: spatial working memory

**Fig. 6:**
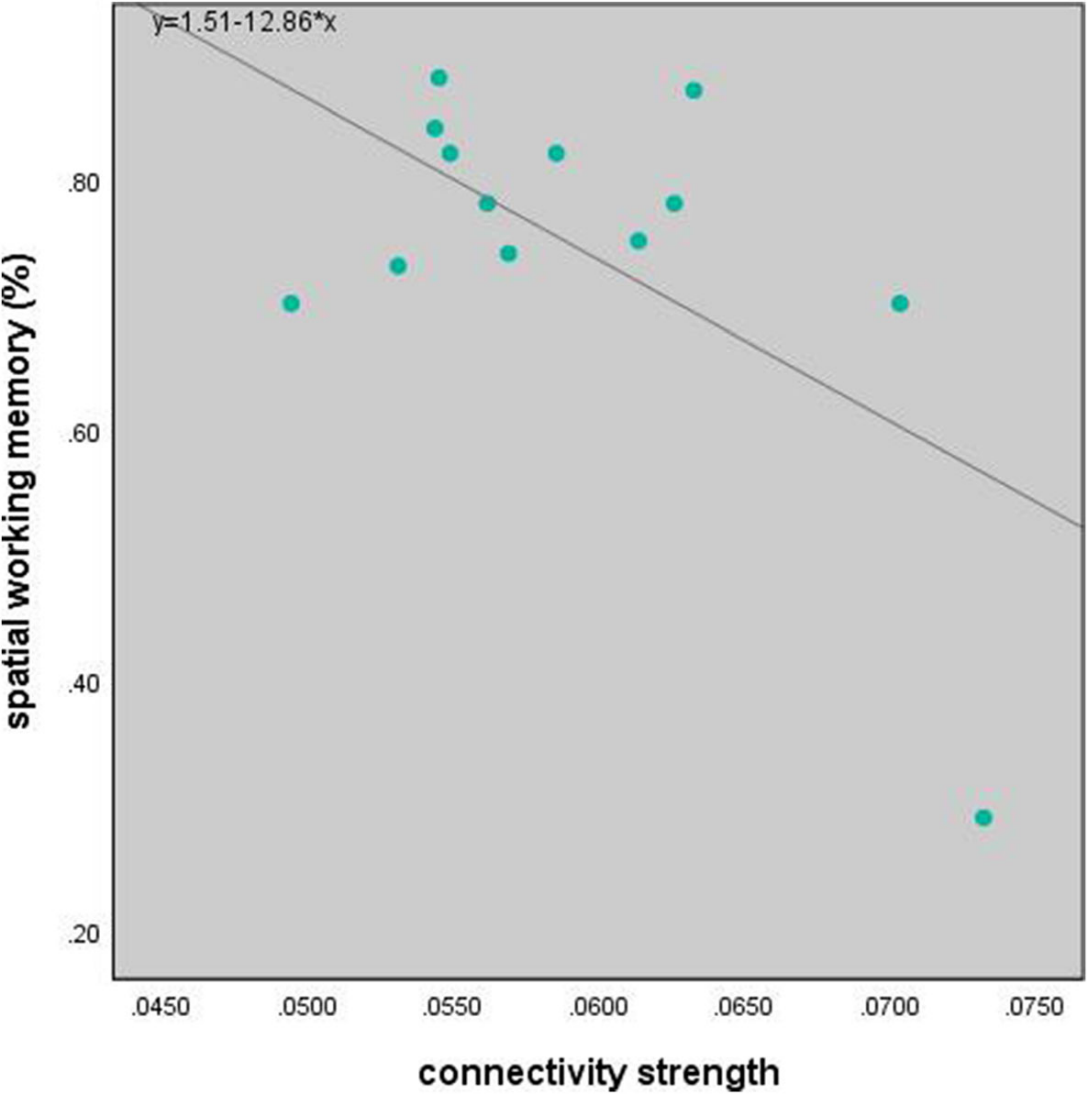
Correlation between SWM and FC of IFG-IPL within left FPN in patients with BP II

To further identified the relationship between working memory accuracy and FCs, Pearson correlation showed that the stronger the FCs’ strength of MFG-IPL and IPL-preSMA, the higher accuracy of SWM task within left FPN in patients with UD (*r* = 0.860, *p* = 0.006; *r* = 0.752, *p* = 0.031; Fig. 5). However, the FC strength of IFG-IPL was negatively correlated with the accuracy of SWM task within left FPN in patients with BP II (*r* = − 0.591, *p* = 0.033; Fig. 6).

## Discussion

Our study showed that the SWM of patients with whether UD or BP II was impaired, but VWM wasn’t impaired. Even in the depressive episode, the patterns of FCs within these two groups of patients were different when performing working memory tasks. The altered FCs of FPN suggest that the IPL may be the key component to distinguish between BP II and UD.

The performances of UD and BP II groups were similar in two working memory tasks, and the accuracy was independent of clinical characteristics. Some research suggested that depression might be associated with insufficient activity of γ-aminobutyric acidergic (GABAergic) neurons which was the main condition for gamma oscillations generation [24–26]. Additionally, human and animal studies reveal that gamma oscillations show a significant reduction in Alzheimer’s disease which is characterized by memory decline [27, 28]. Furthermore, both high and low gamma oscillations were found to be the key to the successful execution of working memory [29, 30]. We hypothesized that depressive individuals may have impaired GABAergic network inhibition mechanisms resulting in gamma oscillations reduction and working memory deficits.

Previous studies demonstrate an increase in both high and low gamma activity in healthy controls during verbal and non-verbal memory tasks [31–33]. However, we found that the deficits of working memory were inconsistent, and only SWM performed abnormally in both UD and BP II patients. Previous researches indicated that negative emotions, such as depression and anxiety disrupted the performance of SWM selectively [34, 35]. From the work of Baddeley and Hitch, they proposed a multi-component model of working memory, the central executive, visuospatial sketchpad, episodic buffer, and phonological loop, and each component had different processing methods to cope with corresponding stimulation [36]. Verbal and spatial location information is processed by different components of working memory. Our tasks of working memory used in this study mainly involve the updating function of the central executive, phonological loop, and visuospatial sketchpad. The phonological loop is equivalent to the buffer which is mainly responsible for the storage and processing of verbal information, furthermore maintains memory accomplished by repetition [37]. Visuospatial sketchpad processes location information by spatial selective attention, which will consume more resources of exogenous attention [38, 39]. Therefore, our study suggested that there might be different components of working memory that was impaired varying degrees in patients with depression, resulting in different performance in two working memory tasks. Besides, depressive patients always have a tendency of rumination which makes them involve negative thoughts deeply [40]. For this reason, when the patients were in depression, cognitive resources were occupied by negative moods and rumination, which made it difficult to put resources into external stimuli. Hence the decline in SWM that requires higher cognitive resources is more obvious. Conversely, based on attentional control theory, we considered that the reason why VWM was complete might be compensatory [41]. When interfered with by emotional factors, individuals will invest more cognitive processing resources to ensure the successful completion of the main task and the same level of homework. In other words, negative emotions are likely to specifically weaken an individual’s ability to work and remember, but this effect is masked by compensatory. When interfered with by emotional factors, individuals would invest more cognitive processing resources to ensure the successful completion of the task. In other words, negative moods might reduce individuals’ accuracy of VWM, but concealed by compensatory.

Next, we will further discuss the neurophysiological mechanisms of working memory deficits in patients. More recently studies provided evidence that working memory impairments appeared to emerge altered gamma oscillatory activity in the frontoparietal regions [42–45]. Furthermore, there was a study that systematically examined markers of GABA signaling across cortical nodes responsible for SWM. SWM is carried out by communication across a distributed cortical network including the primary visual cortex, visual association cortex, posterior parietal cortex, and DLPFC [46]. Inhibition control was considered to be the basic component of the central execution system of working memory. The decline in Inhibition Theory of Hashcr and Zacks believed that the cause of the decreased working memory in the elderly was actually the declining inhibition, rather than the reduction of working memory capacity. Subsequent studies provided evidence to support this theory, moreover found that inhibition was highly correlated with the frontal lobe. Therefore frontal lobe lesions would lead to failed inhibition, then leading to cognitive deficits [47, 48]. However, Redick et al. [49] proposed the opposite theory that the size of working memory capacity could affect the efficiency of inhibition. Both MFG and IFG were considered important components for inhibition and preSMA was also closely related to inhibition. Some findings suggested that bilateral IFG, right MFG, and parietal lobe were co-activated regions when subjects performed inhibition and working memory tasks [50]. The degree of activation in IFG was related to the rate of completion of inhibitive tasks [51]. Whereas Smith and Jonide [52] speculated that the preSMA was responsible for the retelling of spatial information, and activation of preSMA was also be found during SWM performance in the fMRI study. For non-verbal working memory, preSMA exhibited high aggregation with the DLPFC [23]. In short, activation of the prefrontal lobe was abnormally reduced when completing the n-back task [53]. For IPL, as Alain noted, the IPL activation overlapped with that observed during the auditory SWM task [54]. Some studies indicated altered FC of IPL was associated with lower levels of happiness or depression and rumination [55, 56]. IPL was found to play an important role in selective attention, WM rehearsal, and capacity [23, 57, 58]. Apart from this, other results highlighted the role of the IPL in modulating frontal lobe attention network activity [59]. Previously, a resting-state fMRI research in patients with depression showed a lower FC between DLPFC and bilateral posterior parietal cortex, which might directly affect cognitive control function [15]. Hence, we speculated that impaired top-down regulation from the prefrontal lobe might cause damage to the parietal lobe and alteration of the parietal lobe results in SWM deficit.

Our results suggested the patterns of FCs within these two groups of patients were different even performing similarly during working memory tasks. This may be due to the GABA levels evolved differentially between patients with unipolar and bipolar depression. A meta-analysis provides evidence that plasma GABA changes in those with unipolar depression were associated with symptomatic states, whereas plasma GABA changes in those with bipolar disorder seemed to be more closely associated with medication use [60]. Therefore, we believe that the gamma oscillation activity of UD patients would be more affected when they are also in a depressive episode. Additionally, our results also indicated that the FCs of IPL may be a key component to distinguish between BP II and UD. Some brain structural and functional studies have reached similar conclusions. The average kurtosis of gray matter in the IPL could help distinguish bipolar disorder from UD with high accuracy [61, 62]. More recently fMRI study found that UD showed increased fALFF values in the IPL compared to bipolar disorder [63]. Our study provides new evidence to support this conclusion.

## Conclusion

In summary, since the stimulus was presented in the same way, the MSI reflected the endogenous processing of the subjects on both tasks. We demonstrated that only SWM was impaired in both UD and BP II patients. Furthermore, we suggested that the patterns of FCs within these two groups of patients were different even performing similarly during working memory tasks, and IPL may be a key component to distinguish between BP II and UD. Although there were some limitations in our study. Bipolar disorder patients were characterized by increased FC within regions of the FPN [64], which was inconsistent with our results. There was a higher FC strength but not significant between BP II and HC. The reason might be that the size of the participants was too small caused by limited conditions and long duration MSI. In further study, we will include more participants.

## Data Availability

The datasets analysed during the current study are available from the corresponding author upon reasonable request.

## Abbreviations

FPN: Frontoparietal network
DLPFC: Dorsolateral prefrontal cortex
VLPFC: Ventrolateral prefrontal cortex
UD: Unipolar depression
BP II: Bipolar II depression
HAMD: 24-item Hamilton Depression Scale
YMRS: Young Mania Rating Scale
MEG: Magnetoencephalography
MRI: Magnetic resonance imaging
ROI: Regions of interest
MNI: Montreal Neurological Insitute
MFG: Middle frontal gyrus
IFG: Inferior frontal gyrus
preSMA: Pre-supplementary motor area
IPL: Inferior parietal lobule
SPL: Superior parietal lobule
HC: Healthy controls
VWM: Verbal working memory
SWM: Spatial working memory
FC: Functional connectivity
GABA: γ-Aminobutyric acid
